# Advances in the endoscopic diagnosis and treatment of Barrett’s neoplasia

**DOI:** 10.12688/f1000research.6996.1

**Published:** 2016-01-28

**Authors:** Fergus J.Q. Chedgy, Kesavan Kandiah, Sreedhari Thayalasekaran, Sharmila Subramaniam, Pradeep Bhandari

**Affiliations:** 1 Department of Gastroenterology, Queen Alexandra Hospital, Portsmouth, UK

**Keywords:** Endoscopy, Barrett’s neoplasia, Barrett’s oesophagus, oesophageal adenocarcinoma

## Abstract

Barrett’s oesophagus is a well-recognised precursor of oesophageal adenocarcinoma. The incidence of oesophageal adenocarcinoma is continuing to rise in the Western world with dismal survival rates. In recent years, efforts have been made to diagnose Barrett’s earlier and improve surveillance techniques in order to pick up cancerous changes earlier. Recent advances in endoscopic therapy for early Barrett’s cancers have shifted the paradigm away from oesophagectomy and have yielded excellent results.

## Introduction

Barrett’s oesophagus (BO) is defined as the replacement of the normal distal oesophageal squamous epithelium with metaplastic columnar epithelium ^1^. This metaplastic epithelium accumulates genetic changes that over time can progress to dysplasia and cancer ^2^. Screening for BO with endoscopy remains controversial because of poor uptake and lack of cost-effectiveness. A number of alternatives have been proposed to remedy the situation: cytosponge, transnasal endoscopy (TNE) and oesophageal capsule endoscopy (OCE). Surveillance of BO has been demonstrated to diagnose oesophageal cancers earlier and provide a better prognosis ^3^. Surveillance has been improved by advances in endoscope technology and techniques, including high-definition endoscopy and chromoendoscopy. The advantage of detecting oesophageal neoplasia at an early stage is that it can be successfully treated endoscopically without resorting to oesophagectomy.

## Screening for Barrett’s oesophagus

There is a lack of high-quality evidence supporting the use of conventional endoscopy in population-based screening for BO. Ten percent of patients with gastro-oesophageal reflux disease (GORD) have BO ^4^. However, even if screening were performed for every adult with GORD, 40% of oesophageal adenocarcinomas would still be missed ^5^. This has prompted research to identify less invasive and more cost-effective and acceptable methods to screen for BO, such as cytosponge, TNE and OCE.

### Cytosponge

The cytosponge is a sponge contained within a capsule that is attached to a string. The capsule is ingested with water and dissolves in the stomach after 3 to 5 minutes. The string then is pulled to retrieve the sponge and cells collected from the oesophagus ^6, 7^. The cells are analysed with the biomarker trefoil factor 3 to make a diagnosis of BO.

An initial study of the cytosponge ^6^ demonstrated a 3% pickup rate of BO in a primary care setting with a majority of patients (82%) reporting low levels of anxiety, making it a potential tool for mass screening. More recently, Fitzgerald *et al.* followed up on their work with the cytosponge in a large case-control study ^8^. In total, 1,110 patients were recruited: 463 patients with symptoms of dyspepsia and reflux and 647 patients with a prior diagnosis of BO underwent gastroscopy following cytosponge examination; 93.9% of patients had a successful cytosponge examination. Overall, cytosponge sensitivity for detecting BO was 79.9%, increasing to 87.2% in patients with more than 3 cm of BO. The specificity for diagnosing BO was 92.4%. Further trials on the cytosponge device are ongoing but these data suggest that it is an acceptable and accurate device.

### Transnasal endoscopy and oesophageal capsule endoscopy

A recent randomised controlled trial compared the use of unsedated TNE versus sedated gastroscopy for BO screening. Two hundred and nine patients were recruited to standard gastroscopy (surveillance oesophago-gastro-duodenoscopy, or sOGD), unsedated TNE in a mobile research van (muTNE) or a hospital outpatient endoscopy suite (huTNE). Uptake was greater in the unsedated TNE group: 47.5% for muTNE and 45.7% for huTNE versus 40.7% for standard gastroscopy. Complete evaluation of the oesophagus was similar between the groups: 99% muTNE, 96% huTNE and 100% sOGD ^9^.

Chak *et al.* ^10^ examined the acceptability of TNE versus OCE in a randomised controlled trial. They found that uptake for screening examination was low: 15.2% of patients (n = 1,210). Effectiveness of screening for the detection of BO was similar for both technologies. A meta-analysis ^11^ of studies investigating OCE as a screening modality for BO in patients with reflux symptoms demonstrated an overall sensitivity of 77% and a specificity of 86%.

Although technology is advancing in the field of BO screening, there are insufficient data about its cost-effectiveness. These novel approaches appear to be acceptable to patients but more data are required to target whom and when to offer screening.

## Surveillance for Barrett’s oesophagus

In British guidelines the presence of columnar lined oesophagus alone is considered acceptable for a diagnosis of Barrett’s ^1^. American guidelines differ in that they require histological confirmation of intestinal metaplasia to confirm a diagnosis of BO ^12^. Presence of intestinal metaplasia poses a greater risk of neoplastic transformation, and intestinal metaplasia is generally present in longer segments of Barrett’s. The current British guidelines recommend that Barrett’s segments of more than 3 cm have a surveillance OGD every 2 to 3 years and segments of less than 3 cm with the presence of intestinal metaplasia have surveillance OGD every 3 to 5 years. Likewise, the American Society for Gastrointestinal Endoscopy recommends surveillance OGD every 3 to 5 years.

## Endoscopic diagnosis

The annual rate of transformation into oesophageal adenocarcinoma (OAC) in patients with non-dysplastic BO is estimated to be between 0.07% and 0.82% ^13– 15^. However, the annual rate of progression from low-grade dysplasia to high-grade dysplasia (HGD) or OAC is as high as 6.5% ^16– 18^ and from HGD to OAC is 12% to 40% ^19, 20^. Dysplasia in Barrett’s is often flat, patchy and difficult to detect. British Society of Gastroenterology (BSG) guidelines ^1^ recommend the Seattle biopsy protocol, which entails four-quadrant random biopsies every 2 cm in addition to targeted biopsies on macroscopically visible lesions. This surveillance method has had a poor uptake amongst endoscopists as it is time-consuming, labour-intensive, and prone to sampling error ^21, 22^.

Simple techniques such as mucolytic agents and increased inspection times can be used in order to improve visualisation of Barrett’s mucosa during surveillance. N-acetylcysteine is a mucolytic agent that can be used at a concentration of 4% to 10% to dissolve excess mucus and bubbles. Basford *et al.* ^23^ recently reported on a randomised controlled trial (n = 126 patients) comparing a combination of simeticone and N-acetylcysteine (NAC) (group A) as a pre-drink prior to gastroscopy with water alone (group B) and no pre-drink. They reported significant improvement in mucosal visibility with simeticone and NAC as compared with just water or no pre-drink. This pre-drink was also reported to reduce the number of additional flushes to achieve satisfactory views. There is also evidence, in a similar way to colonoscopy, that the longer the duration spent assessing BO, the greater the detection rate for neoplasia ^24^. The study suggests spending at least 1 minute per centimetre of Barrett’s. However, this study was performed in a high-risk tertiary referral population in whom the index of suspicion of neoplasia was high, and therefore may not apply to the routine surveillance population.

### High-definition white light endoscopy

With the advent of charge-coupled device (CCD) chips, high-definition white light (HDWL) endoscopes are able to capture and display high-definition images with pixel densities of more than 10 million pixels, making standard definition (pixel density of 100,000 to 400,000) endoscopes obsolete ^25^.

The sensitivity and specificity of HDWL endoscopy in detecting Barrett’s neoplasia are 40%–64% and 98%–100%, respectively ^26, 27^. BSG and American Gastroenterological Association (AGA) guidelines recommend the use of high-resolution endoscopes for Barrett’s surveillance ^1, 28^.

### Virtual chromoendoscopy

The enhancement of mucosal surface and vascular patterns using optical and digital filter technologies has added to the arsenal of the advanced endoscopist in the quest to improve dysplasia detection. Narrow band imaging (NBI) (Olympus, Tokyo, Japan) and blue laser imaging (BLI) (Fujifilm, Tokyo, Japan) use a filter located in front of the light source. This technology filters white light and limits the wavelength of the light projected to 415 to 540 nm ^29^. When projected onto a mucosal surface, this ‘narrow band’ of light appears blue and green. The blue light penetrates the superficial layer of the mucosa, thereby enhancing the view of superficial capillaries and the crypt patterns in the mucosal surface. In contrast, technologies such as i-Scan (Pentax, Tokyo, Japan) and Fujinon intelligent chromoendoscopy (FICE) (Fujinon, Tokyo, Japan) employ complex proprietary algorithms to digitally reproduce a narrow-spectrum image at the push of a button on the endoscope. BLI ^30^ is a new technology with white and blue lasers that produce narrow-band light.

***Narrow band imaging.*** NBI is the most studied optical imaging technology thus far and has a sensitivity and specificity of 47%–100% and 72%–100%, respectively, for detecting Barrett’s neoplasia ^27, 31– 34^. NBI selectively enhances mucosal vascular patterns by narrowing the spectrum of light, reducing the amount of red light in the displayed image whilst narrowing the spectrum of blue and green, making blood vessels appear dark against the background mucosa ^35^.

A majority of these studies were conducted in tertiary referral centres by expert endoscopists evaluating an enriched population with a high index of suspicion of neoplasia. The technology has yet to be validated in non-expert hands or in a surveillance population. Therefore, we would suggest that training in the use of this technology and data in a surveillance population be required prior to adoption in routine clinical practice.

***i-Scan.*** i-Scan uses post-processor technology that reconstructs the image transmitted from the endoscope by using a computer-based algorithm which is able to accentuate both surface patterns and vasculature ^35^. A randomised control trial found that the yields of acetic acid-guided versus i-Scan-guided biopsies in detecting specialised columnar epithelium were comparable ^36^. Verna *et al.* found that dysplasia detection rate using this technology was inferior to that using the standard four-quadrant biopsy technique ^37^. However, these studies were poorly designed and have small sample sizes. More robust studies on the utility of i-Scan in the detection of dysplasia in BO are required.

***Fujinon intelligent chromoendoscopy.*** FICE uses a CCD in the endoscope to capture spectral reflectance data. A matrix processing circuit found in the video processor then receives the data. The reflectance spectra of corresponding pixels that make up the conventional image are mathematically estimated. From this information, a single-wavelength virtual image is reconstructed. Three such single-wavelength images can be selected and assigned to the red, green and blue monitor inputs to display a composite colour-enhanced multiband image in real time. This can be used like NBI to remove data from the red part of the waveband and narrow the green and blue spectra ^35^.

There is a paucity of research evaluating the utility of FICE in detection of dysplasia in Barrett’s. The sole published study to date is a prospective pilot study carried out in a tertiary centre. It was found that the dysplasia detection rate of FICE, when used in conjunction with acetic acid, is 86% ^38^.

***Autofluorescence imaging.*** Autofluorescence imaging (AFI) is based on a principle that a specific light wavelength can cause fluorescence of endogenous biomolecules such as collagen, nicotinamide adenine dinucleotide (NADH), flavin adenine dinucleotide (FAD), and porphyrins. These molecules can accumulate in dysplastic oesophageal mucosa ^39^. A randomised cross-over multi-centre trial on an enriched population found a marginal gain of AFI over quadrantic biopsies which did not reach statistical significance ^40^. Endoscopic trimodal imaging (ETMI) systems which integrate AFI with HDWL and NBI have not been shown to be superior to standard resolution white light endoscopes ^41, 42^. With a lack of evidence of its efficacy and high false-positive rates, the use of AFI and ETMI at present remains in the domain of endoscopic research ^39– 41^.

### Chromoendoscopy with white light endoscopy

***Methylene blue chromoendoscopy.*** Three randomised cross-over trials found that the diagnostic accuracy of methylene blue 0.5%-directed biopsies is higher than random 2 cm quadrantic biopsies ^43– 45^. However, a meta-analysis which included data from six trials found that overall methylene blue chromoendoscopy was not superior to random biopsies in the detection of specialised intestinal metaplasia or dysplasia ^46^.

**Figure 1.**
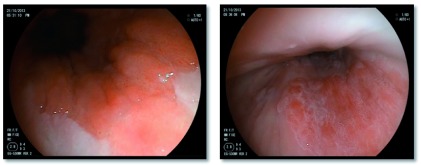
Intramucosal carcinoma (left) and high-grade dysplasia (right) highlighted by acetic acid.

***Acetic acid chromoendoscopy.*** Acetic acid is a weak acid that causes acetowhitening of the oesophageal mucosa. Over a period of seconds to minutes, dysplastic tissue will start to lose the acetowhitening effect before the surrounding non-dysplastic Barrett’s tissue. Differential loss of acetowhitening highlights the neoplastic focus as a red spot on a white background ( Figure 1). This is an extremely promising technique with high sensitivity, universal applicability and negligible cost.

Two large cohort studies have demonstrated effectiveness of acetic acid used at concentrations of 2.5% and 1.5%, respectively ^47, 48^, in the detection of dysplasia within Barrett’s in high-risk populations. The two reported similar results, with sensitivities for dysplasia between 90% and 95% and specificities between 75% and 85%. A further study using 2.5% acetic acid found that the number of biopsies needed to detect neoplasia could be significantly reduced if acetic acid targeted biopsies were used in place of mapping biopsies, thereby reducing pathology-related costs by 97% ^49^. Tholoor *et al.* ^50^ reported the use of 2.5% acetic acid in a surveillance population and were able to demonstrate a threefold increase in neoplasia detection as compared with conventional protocol-guided biopsies. However, this was a non-randomised trial and an ongoing randomised trial, the ABBA study ^51^, will answer this question soon. The ABBA study is a randomised, crossover, tandem endoscopy study comparing standard quadrantic biopsy protocol versus acetic acid targeted biopsies, in a Barrett’s surveillance population.

***Cross-sectional optical imaging.*** Optical coherence tomography (OCT) and confocal laser endomicroscopy (CLE) are emerging technologies that are able to obtain micro-anatomical images of the oesophageal mucosa. OCT technology measures the difference between the backscatter of near-infrared low-coherence light below the tissue surface and a reference beam ^52^. Using this information, it is able to reconstruct the microanatomy of the superficial mucosal layer ^53^. CLE, on the other hand, involves the integration of a confocal laser microscope in the distal tip of a conventional video endoscope ^54^. Although both technologies have sensitivities of 68% to 86% and specificities of 73% to 83% ^55, 56^, the setup costs are high and special training is required for image interpretation. This currently limits its use in routine clinical practice. Table 1 summarises the performance of current imaging technologies in the diagnosis of Barrett’s neoplasia.

**Table 1.** Performance of current imaging technologies in the diagnosis of Barrett’s neoplasia.

Imaging technology	Sensitivity	Specificity	References
High-definition white light	40%–64%	98%–100%	26, 27
Narrow band imaging	47%–100%	72%–100%	27, 31– 34
Autofluorescence imaging	42%–50%	61%–92%	39, 73
Methylene blue	49%–51%	48%–85%	44, 45
Acetic acid	90%–95%	75%–85%	47, 48
Optical coherence tomography	68%–83%	75%–82%	55, 74
Confocal laser endoscopy	68%–86%	83%–88%	56

## Endotherapy

Until recently, oesophagectomy was regarded as the gold standard treatment for patients with HGD or early oesophageal cancer ^57^. Even in expert hands, oesophagectomy carries significant morbidity and mortality: 30% to 50% and 2% to 5%, respectively ^58– 60^. Not only are surgical risks high but the patient population with this condition have co-morbidities that often preclude surgical intervention. In the last 15 years, endoscopic therapy has become an established treatment of HGD and intramucosal adenocarcinoma (T1a) as the risk of lymph node metastases is very low ^1^.

### Endoscopic mucosal resection

**Figure 2.**
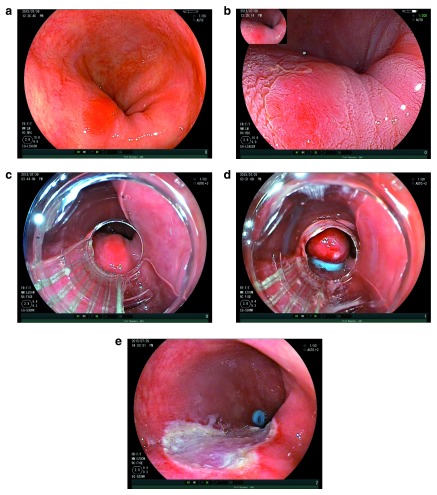
EMR of Barrett’s HGD. ( **a**) An area of Barrett’s high-grade dysplasia. ( **b**) The same area demonstrating acetowhitening effect. ( **c**) The same lesion as viewed down a multi-band ligator. ( **d**) Pseudopolyp created by the band ligator. ( **e**) Resection defect following endoscopic mucosal resection.

Experience of endoscopic resection (ER) for early oesophageal adenocarcinoma began in the early 1990s in Asia; since then, techniques have significantly progressed. Initial experience came from the strip biopsy technique, which was further refined by the suck-and-cut and multi-band ligator techniques ( Figure 2) ^61^. Ell *et al.* ^62^ reported the first (n = 64) series of ER for early Barrett’s cancer, demonstrating a 97% remission rate. Despite a short follow-up period (mean of 12 months), there was a significant rate of recurrence of 14%.

In the early years, a high rate of metachronous and recurrent lesions hampered the apparent technical success of ER with reported recurrence rates of up to 35% ^52^. This led to a refinement of techniques incorporating both endoscopic mucosal resection (EMR) and ablative therapies (discussed below). A randomised trial ^63^ (The APE Study) comparing EMR + argon plasma coagulation (APC) ablation versus EMR + observation demonstrated a significantly reduced risk of metachronous lesions in the ablation arm, 3% versus 37%, such that ablation following ER is now standard treatment.

Pech *et al.* ^52^ reported their outcome data of 1,000 patients with early Barrett’s cancer treated by ER, demonstrating a long-term complete remission rate of 93.8%. There was a 14.5% recurrence rate, and a majority of recurrences were treated endoscopically. Their serious complication rate was 1.5%, and no mortality was reported.

## Ablative therapies

Given the recognition that ablation following ER offers optimum outcomes in terms of neoplasia eradication, it now forms an essential component of the treatment pathway of early Barrett’s cancer. The two main techniques are APC and radiofrequency ablation (RFA). The findings of the APE study ^63^ are described above. A recent meta-analysis ^64^ of RFA has shown intestinal metaplasia eradication rates of 78%, dysplasia eradication rates of 91%, and cancer progression rates of 0.2% to 0.5% with an oesophageal stricture rate of 5%. Data from the UK RFA registry ^65^ report similar rates of success with a complete dysplasia remission rate of 92% and a stricture rate of 6.2%.

There are currently no data comparing outcomes of APC and RFA to determine which is more effective. However, the Barrett’s Intervention for Dysplasia by Endoscopy (BRIDE) study aims to answer this question. Whilst RFA is an extremely effective treatment, its costs remain high (up to £1,800 per catheter), and EMR followed by intensive acetic acid surveillance has been shown to produce outcomes similar to those of the UK RFA registry at a significantly cheaper cost ^66^.

Early experience with cryotherapy shows promising results with dysplasia eradication rates of up to 97% in patients with short-segment BO ^67^. This technology, however, remains firmly in the research domain until further data are available.

### Endoscopic submucosal dissection

**Figure 3.**
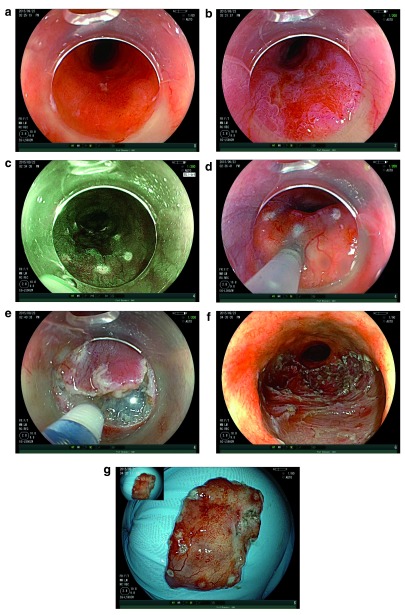
ESD of Barrett’s IMC. ( **a**) pT1a/M3 intramucosal cancer arising in Barrett’s oesophagus. ( **b**) The same lesion following acetic acid. Note the differential early loss of acetowhitening. ( **c**) Edges of the lesion marked with endoscopic submucosal dissection (ESD) knife under virtual chromoendoscopy. ( **d**) Submucosal injection. ( **e**) Mucosal incision with ESD knife. ( **f**) Resection base following ESD. ( **g**) Resection specimen.

The main limitation of the EMR technique is that en bloc resection is possible only for lesions of less than 15 mm; lesions larger than this require piecemeal resection, making adequate histological assessment difficult. Figure 3 demonstrates the steps involved in the endoscopic submucosal dissection (ESD) technique. Experience with ESD in Japanese studies of early oesophageal squamous cell cancer has demonstrated improved outcomes over EMR. To date, three European studies have reported outcomes of ESD for neoplastic Barrett’s. Neuhaus *et al.* ^68^ reported on 30 patients with early neoplastic lesions up to 30 mm: en bloc resection was achieved in 90%, the complete neoplasia eradication rate was 96.4%, and there were no reported complications. More recently, Chevaux *et al.* ^69^ reported on their outcomes of 75 consecutive patients; ESD was performed on lesions of more than 15 mm, achieving an en bloc resection rate of 90% and a neoplasia eradication rate of 92%, and oesophageal strictures developed in 60% of patients. Probst *et al.* ^70^ reported on 87 patients with early oesophageal adenocarcinoma achieving a 95.4% en bloc resection rate with a recurrence rate of 2.4%; 11.7% of patients had stricturing, and no perforations were reported. Disappointingly, however, these studies have reported low R0 resection rates (38.5% to 48.5%).

ESD appears to be a promising addition to current treatment techniques, especially for larger lesions. Our own data on 51 ESDs, on selected patients with Barrett’s cancer, have demonstrated an R0 resection rate for cancer of 88%, a recurrence rate of 3% and no complications ^71^. A recently reported randomised controlled trial of EMR (n = 20) versus ESD (n = 31) demonstrated superior en bloc, R0 and curative resection rates for ESD; however, there was no difference in clinical outcome for either technique ^72^. In our experience, large nodular lesions have a high risk of containing cancer and therefore we believe they should be removed in an en bloc fashion. Early European data have shown the feasibility and safety of ESD in Barrett’s neoplasia but have not proven its superiority over EMR. This has to be addressed in a well-designed study for a select group of patients to identify the exact role of ESD in Barrett’s neoplasia.

## Conclusions

At present, there is insufficient evidence to advocate population-based screening, and novel techniques, though acceptable to patients, have yet to be proven cost-effective. Inevitably advances in endoscope technology will improve dysplasia detection, but we believe formalised training programmes are required to extrapolate trial evidence from expert endoscopists into everyday practice. Outcomes of endotherapy for early Barrett’s neoplasia are excellent and should be considered first-line treatment in this group. Increasing experience of ESD in the West will enable en bloc resection of larger and more advanced lesions with good outcomes, but further trial data are required to clarify who benefits most from this technique.

## Abbreviations

AFI, autofluorescence imaging; APC, argon plasma coagulation; BLI, blue laser imaging; BO, Barrett’s oesophagus; BSG, British Society of Gastroenterology; CCD, charge-coupled device; CLE, confocal laser endomicroscopy; EMR, endoscopic mucosal resection; ER, endoscopic resection; ESD, endoscopic submucosal dissection; ETMI, endoscopic trimodal imaging; FICE, Fujinon intelligent chromoendoscopy; GORD, gastro-oesophageal reflux disease; HDWL, high-definition white light; HGD, high-grade dysplasia; huTNE, unsedated transnasal endoscopy in a hospital outpatient endoscopy suite; muTNE, unsedated transnasal endoscopy in a mobile research van; NAC, N-acetylcysteine; NBI, narrow band imaging; OAC, oesophageal adenocarcinoma; OCE, oesophageal capsule endoscopy; OCT, optical coherence tomography; RFA, radiofrequency ablation; sOGD, surveillance oesophago-gastro-duodenoscopy; TNE, transnasal endoscopy.
